# Hemorrhage after the Extraction of Three Molar Teeth

**Published:** 1859-08

**Authors:** Thos H. Harding

**Affiliations:** Park-square, Regent’s Park


### HEMORRHAGE AFTER THE EXTRACTION OF THREE MOLAR TEETH.

**To the Editor of the London Lancet.**

Sir—I was lately called to see a case of hemorrhage after extraction of teeth, the source of which was evidently the dental artery at the bottom of the sockets; and as it possesses some interest from the fact that at one time it threatened to be hazardous to the life of the patient, I beg to forward a few particulars of it for insertion in your Journal:

A fine, remarkably healthy-looking little girl, twelve years of age, and not at all nervously inclined, had three molar teeth extracted by a friend who holds a first-rate position in our profession. These were a molar in the lower jaw on the the right side, one on the left side, and one in the upper jaw on the left side, being the first permanent teeth. The extraction was accomplished at half-past two o'clock in the afternoon, and followed by arterial bleeding from the sockets of the two teeth from the left side, upper and lower jaw. This continued uninterruptedly till half-past nine o'clock, when I was sent for to see her. The source of the bleeding appeared to me to arise from the small dental artery at the bottom of the socket. The blood was of a bright scarlet color, and continued to flow without cessation. I removed all coagulation from the cavities of both these teeth, and plugged them with cotton-wool saturated with muriated tincture of iron and tannin, which had the effect of most completely controlling the bleeding. I staid an hour with the patient, and then left her without any recurrent hemorrhage. About two o'clock in the morning I was hurriedly sent for, as the bleeding had returned in the same teeth during the night. On my arrival I found the plugs had become disturbed, and were compressed into the sockets by the teeth, but in such a way as to be ineffectual in preventing bleeding. They were therefore removed and the cavities were a second time very firmly stopped with cotton-wool, saturated in the same fluid as before. This so effectually controlled the bleeding that there was no further appearance of it. A gentleman, however, remained with her till six o'clock, to see that no mischief should ensue; for, I may state, the amount of blood lost was very considerable, and had greatly alarmed the child's mother, particularly as she was her only daughter. The next day I prescribed a lotion to rinse the mouth out every quarter of an hour, consisting of alum half a drachm; tannin, ten grains; tincture of myrrh, half an ounce; camphor mixture, seven ounces and a half: and latterly I brushed her gums with a solution of sulphate of zinc, ten grains to an ounce of water; and to take a fourth part of a Seidlitz powder every three hours in four ounces of water. I may say she is now quite well.

I may observe that the teeth were skillfully removed by my friend, and it was necessary to do so, as they were much decayed; and I thought it proper to publish these few particulars, to show that sometimes very troublesome hemorrhage will follow the extraction of a tooth when the hemorrhagic diathesis prevails. The removal of a tooth has actually cost the life of the patient, but in that class of persons a mere scratch or a very slight wound will prove fatal.

I remain, sir, yours very obediently,

THOS. H. HARDING.

Park-square, Regent's Park, 1859.

